# The hydraulic mechanism in the hind wing veins of *Cybister japonicus* Sharp (order: Coleoptera)

**DOI:** 10.3762/bjnano.7.82

**Published:** 2016-06-23

**Authors:** Jiyu Sun, Wei Wu, Mingze Ling, Bharat Bhushan, Jin Tong

**Affiliations:** 1 Key Laboratory of Bionic Engineering (Ministry of Education), Jilin University, Changchun, 130025, P. R. China; 2 Nanoprobe Laboratory for Bio- & Nanotechnology and Biomimetics (NLB2); The Ohio State University, 201 W. 19th Avenue, Columbus, OH 43210-1142, USA; 3 State Key Laboratory of Automotive Dynamic Simulation, Jilin University, Changchun 130022, China

**Keywords:** bioinspiration, diving beetles, hydraulic mechanism, wings, micro air vehicles (MAVs)

## Abstract

The diving beetles (Dytiscidae, Coleoptera) are families of water beetles. When they see light, they fly to the light source directly from the water. Their hind wings are thin and fragile under the protection of their elytra (forewings). When the beetle is at rest the hind wings are folded over the abdomen of the beetle and when in flight they unfold to provide the necessary aerodynamic forces. In this paper, the unfolding process of the hind wing of *Cybister japonicus* Sharp (order: Coleoptera) was investigated. The motion characteristics of the blood in the veins of the structure system show that the veins have microfluidic control over the hydraulic mechanism of the unfolding process. A model is established, and the hind wing extending process is simulated. The blood flow and pressure changes are discussed. The driving mechanism for hydraulic control of the folding and unfolding actions of beetle hind wings is put forward. This can assist the design of new deployable micro air vehicles and bioinspired deployable systems.

## Introduction

The concept of a micro air vehicle (MAV) was first introduced in the early 1990s. It was extensively researched because of its advantages over traditional aircraft, such as its small size, light weight, good concealment, flexibility, low cost, and portability. There are three main flight modes: fixed wing, rotor, and flapping. Insects possess a remarkable ability to fly, far superior to what humans achieved in the production of MAVs with a low Reynolds number.

In general, the hind wings of a beetle are larger than its forewings (elytra) to maintain the ability of the beetle to fly. One exception are the hind wings in perfectly flying jewel beetles, which are not folded at all and are smaller than its elytra. The hind wings of a beetle are membranous and folded under the elytra while at rest [1]. In order to be completely covered by the elytra, the hind wings have to be folded under them, even insofar as to be folded four times. The folding lines found covering the hind wings allow them to be folded in a particular direction. The folding line intersection points and the angle between the folding lines determine the folding pattern [2]. The folding of the hind wings provides the following functionality: (1) flapping wings can change shape, giving them better aerodynamic characteristics [3]; (2) pleated wings are more rigid in flexion than planar ones; (3) when insects are not flying, the folding structure allows the hind wing to be folded as a small package, tucked under the elytra [4]; (4) by a complex folding pattern, the hind wing can be folded to even one-tenth the unfolded size [5]. Aside from muscle tissue, which can control several veins near the wing base, there are no supporting elements, such as bones or muscles, in the hind wing itself, and the folded region is at the tip of the wing. How, then, does a beetle realize the folding/unfolding of its hind wings? It was generally believed that this was achieved by the combined effect of external forces (wing base, thoracic muscle) and vein characteristics (such as vein discontinuities, venation change, and vein membrane elasticity) [1]. Additionally, the hemolymph runs within the veins, assisting in the folding and unfolding movements of the wings [5].

The folding/unfolding behavior of beetle hind wings has been extensively researched. Its mechanism has been under continuous investigation [3]. Forbes [6] first investigated the folding process of the hind wings of a beetle (*Pachnoda marginata*). When beetles are at rest, their wings will be folded and tucked under the elytra, and the folding process includes the lateral and longitudinal folding of the membranous hind wings. Folding is carried out along a folding line [7]. It may involve longitudinal folding of the wing membrane and sometimes transverse folding [1,8–9]. The beetle hind wing folds in a four-panel mechanism, meaning that the hind wing has four hinged plates as the basic structure, which are mutually folded when combined [10–12]. The system has a single degree of freedom of motion, which is composed of four folding lines connecting the four plates. The folding and unfolding is achieved by decreasing and increasing the angle between the two plates. In fact, integration of some basic mechanisms produces the various folding patterns [11]. Moreover, the hind wing diamond area type can play a central role as a spring [11,13]. The ribs are arranged in a certain way.

Another possibility is that the folding and unfolding actions are controlled by two different mechanisms [14]. Folding requires the synergistic action of abdominal and thoracic muscle forces [15–16]; resilin in some mobile joints, together with data on wing unfolding and flight kinematics that may result in elastic energy storage in the wing [17]; or leveraging the rigid wing membrane involved in the folding action [18]. The unfolding action comes from the contraction of muscles [1,8].

An insect wing consists of a thin membrane and a system of veins. There are cavities within major veins that contain nerves and trachea, and because they are connected with the hemocoel, hemolymph can flow into the wings (http://medlibrary.org/medwiki/Insect_wing). Hemolymph can transfer mechanical pressure caused by muscle contraction, and facilitate fluid-feeding, prey capture, pupation, and the ecdysis and eclosion processes [19]. However, this has not been confirmed or rejected by experiments regarding unfolding or folding actions [2].

In a previous work [20], we investigated the various hydraulic forces for the unfolding process of the hind wings in *Dorcus titanus platymelus* (Lucanidae, Coleoptera), which is a xylosaprophagous beetle, and its hind wings are folded down to 55% of their full length. In this paper, the hydraulic mechanism in the veins of hind wings of diving beetles (*C. japonicus*) was investigated, and the unfolding process, including blood flow and pressure changes, was simulated. The study of the hydraulic mechanism in the folding and unfolding process will be provide insights for the design of micro air vehicles with morphing wings, and give inspiration for the development of bioinspired deployable systems.

## Experimental

### Beetles

**Figure 1:**
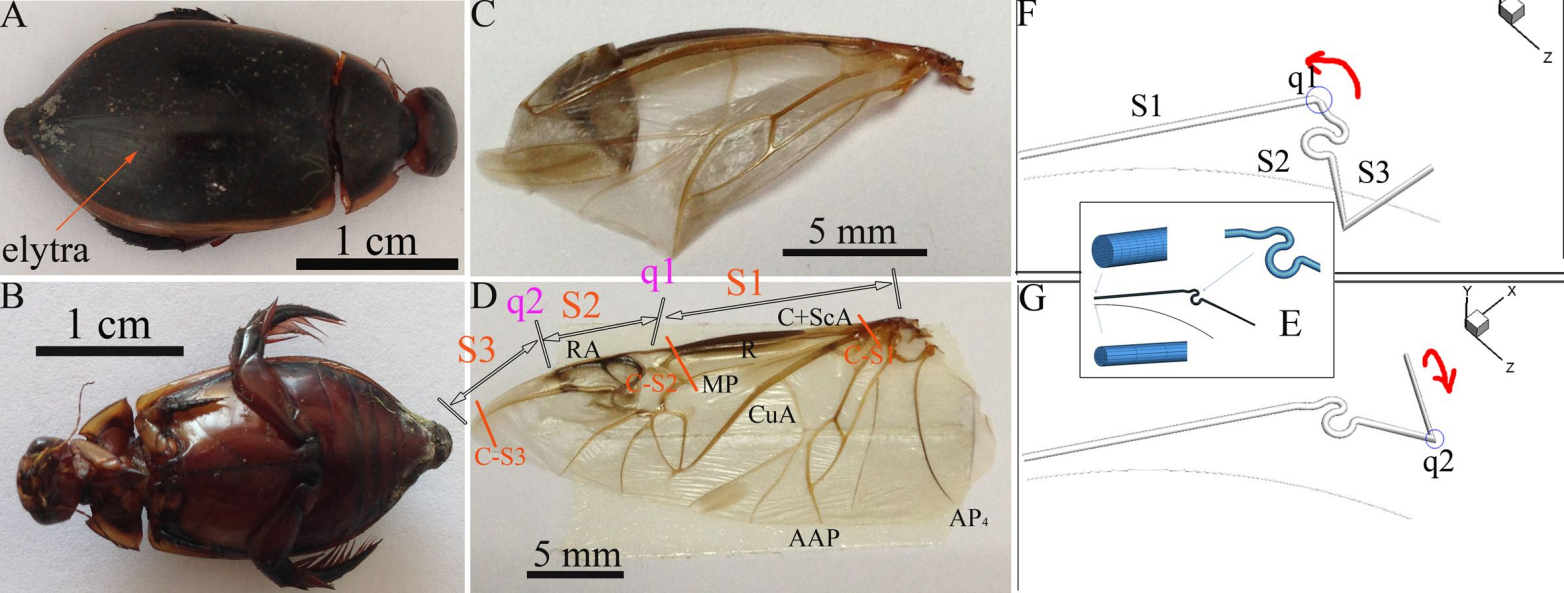
(A) and (B) *C. japonicus*, excised hind wings in folded state (C) and unfolded state (D), where C is costa, ScA is subcosta anterior, RA is radius anterior, R is radius, MP is media posterior, CuA is cubitus anterior, AAP is anal anterior posterior, and AP_4_ is anal posterior. (E–G) The local mesh figure and the vein movement diagram. The costa vein was set as three segments: S1 is fixed, S2 is connected to S1 and can rotate, and S3 is connected to S2 and can rotate. S1 simulates C+ScA, while S2 and S3 simulate RA, which is the folded vein. q1 and q2 are rotating points. C-S1 to C-S3 are the cutting positions of the cross sections of the wing base, the posterior part of the wing, and the folded zone in C for Figure 3.

The diving beetle (*C. japonicus*) is an aggressive predator: larvae and imagines devour small fish and invertebrates (Figure 1A and Figure 1B). They live in fresh water and can swim actively. They have a pronounced flight capability when leaving the water, and they fly to migrate from one body of water to another. Wings are folded and the elytra are closed during the imaginal life activities of the beetle (swimming, hunting, reproduction), not only during rest. Their hind wings are folded under the elytra when at rest. Figure 1C shows an excised hind wing in the folded state, in which it is 30% shorter than in the deployed state (Figure 1D). The venation is shown in Figure 1D, where C is costa, ScA is subcosta anterio, RA is radius anterior, R is radius, MP is media posterior, CuA is cubitus anterior, AAP is anal anterior posterior, and AP_4_ is anal posterior. Figure 1E–G shows the simulation model of a folded vein of *C. japonicus*. For observation and experimentation, beetles were captured in the wild in Guangdong City, Guangdong Province, China. Their body length was 35–40 mm.

### Microstructure testing

Microstructure testing was conducted in a manner similar to that described in [20]. The microstructures of the cross sections of hind wing veins were captured using an inverted fluorescence microscope (OLYMPUS, LX71).

All beetles were anesthetized with ether. Their hind wings were cut out with a scalpel, and a 1 mm^2^ area was sliced from the wing base, folded zone, and the posterior part of the wings. These were then quickly dipped in a 20% epoxy resin 812 solution. After cooling in ambient environment, they were put into an embedding box. Dehydration was reached with a gradient concentration of ethanol and acetone to replace the water in the wing tissues (using 70%, 80%, and 95% ethanol one time each for 10 min; 95% ethanol and 95% acetone (1:1) for 10 min; 100% concentration acetone twice for 20 min; and 100% concentration propylene oxide once for 20 min). After that, ultrathin sections of 50–100 nm thickness were cut out by an ultrathin slicing machine. In order to easily view the sample structure, dye (hematoxylin eosin) was used to stain the specimens.

To confirm which veins of the hind wings were involved in the hydraulic mechanism during unfolding, a retinal camera (Topcon, TRC-50DX-Type IA) was used. Retinal camera testing was performed with a fluorescent agent to color the walls of the veins. One milliliter of green fluorescent indicator (FITC) was injected into the beetle abdomen at time zero. The measurement was taken with an excitation wavelength of 488 nm in the retinal camera.

The unfolding hind wings process of the hind wings of a flying beetle was photographed with a high-speed camera (OLYMPUS, *i*-SPEED 3, camera speed of 400 frames/s). The beetle was suspended in front of the camera.

A biological pressure sensor and dynamic signal acquisition and analysis (DSA) were designed as a control system to investigate the variation of fluid pressure in the veins of the hind wings [20].

### Definition of FLUENT software parameters

The speed and pressure changes during the unfolding process were numerically simulated via computational fluid dynamics (CFD) solver. To simulate the insect wing veins within the fluid flow during the unfolding process, the CFD solver, FLUENT 6.3.26, was used to solve the momentum conservation equations (Navier–Stokes equation, NS equation) based on the pressure method. The motion of an unfolding wing was modeled by using the dynamic mesh technique.

Assuming that the flow is laminar, the fluid medium is blood, and the inlet effect is not considered, the control equation of fluid flow in the beetle hind wing is the 3-D incompressible NS equation:

[1]
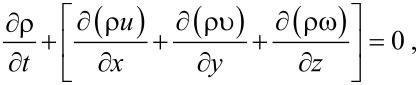


where *u*, *v*, *w* are the velocity components (m/s) for the *x*, *y*, *z*-directions, respectively, and ρ is the density of the fluid. Momentum equations are given as,

[2]
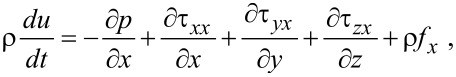


[3]
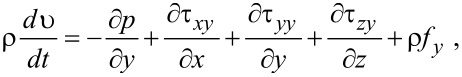


[4]
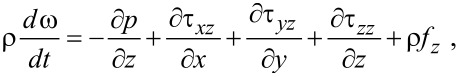


where *f_x_*, *f_y_* and *f_z_* are the generalized source terms for the momentum equation, only for inertia force, the external forces on the volume element in the *x*, *y* and *z*-directions *F_x_* = *F_y_* = *F_z_* = 0.

The purpose of simulation in this is the analysis of the motion of veins, mainly focusing on the vein expansion process, and the variation of the flow field and coordination. Thus, when defining the parameters for the simulation study, the model was set to a rigid body. If we are only concerned about the whole movement of the object and the internal deformation does not affect the whole movement, then the object can be simplified as a rigid body with no penetration of the wall [21].

## Results and Discussion

### Experimental

**Figure 2:**
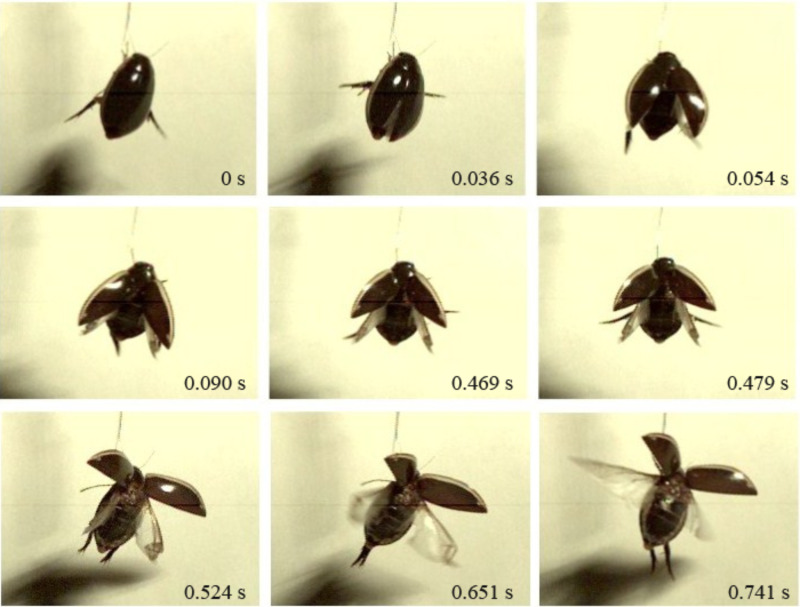
The unfolding process of the hind wings of *C. japonicus* captured by a high-speed camera.

The deployment process of the hind wings of *C. japonicus* was recorded using a high-speed camera, as shown in Figure 2. At 0.036 s, the opening of the elytra appears; at 0.054 s, the hind wings slip from the elytra that are gradually rotating and ascending to a certain height; at 0.651 s, the hind wings start flapping but are still folded; until 0.741 s, the elytra keep rotating outward and lifting, creating sufficient space for the hind wings to flap. It is obvious that the hind wings are still folded at this time (0.741 s), and the double wings flap down with almost no time difference. Live beetles can control the state of wing folding by coordinated, sometimes asymmetrical or repeated movements of the hind wings, elytra, prothorax, and abdomen [22]. In particular, when longitudinal muscles contract, the tergum rises upwards with respect to the pleura (the fulcra of the wings) and thus move both wing plates down by the lever principle [23]. For air dynamics, the hind wings flapping at the same time helps to obtain aerodynamic force, resulting in a successful take-off [15–16,24–25]. At 0.759 s, the hind wing movement is at its lowest point, and the hind wings are completely expanded.

**Figure 3:**
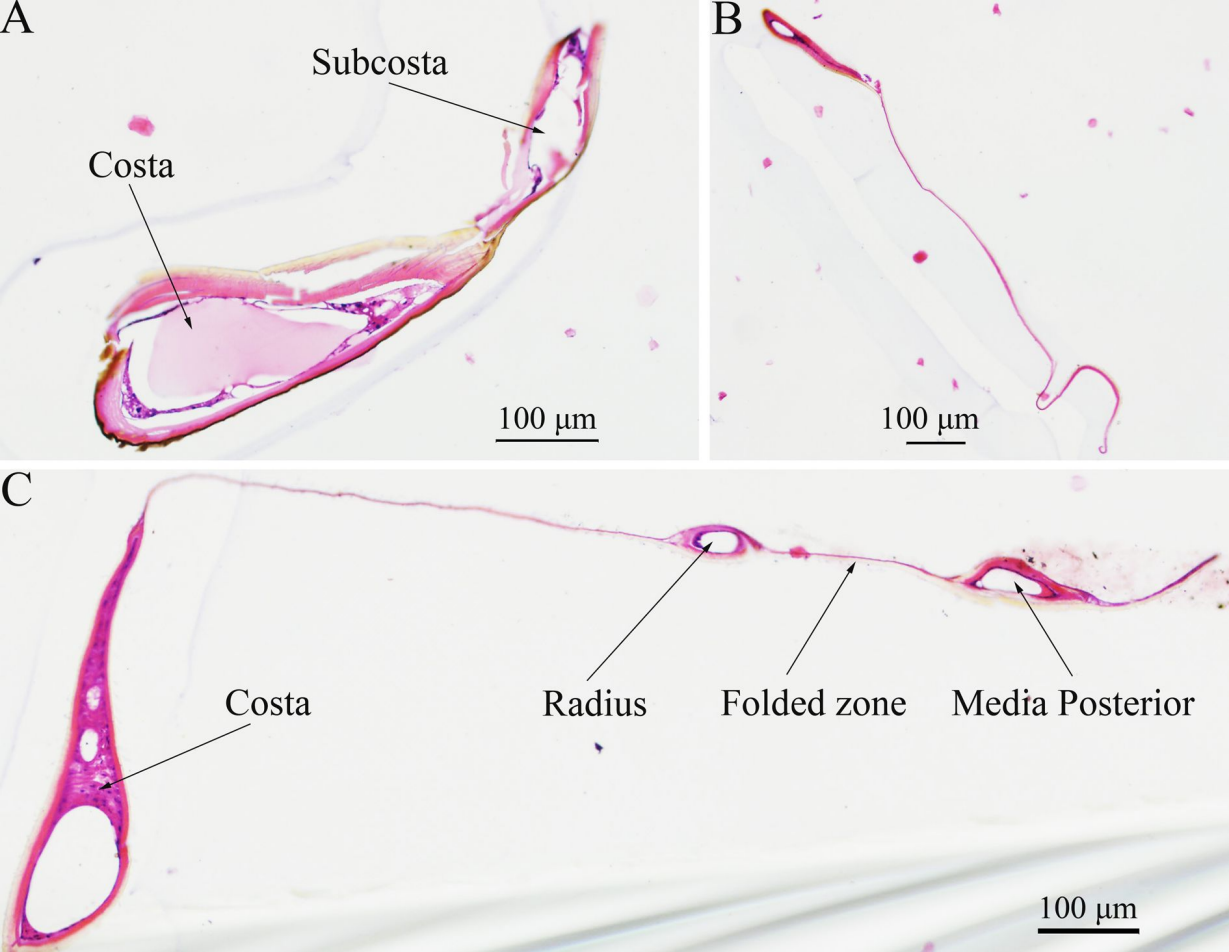
The cross sections of (A) the wing base (C-S1), (B) the posterior part of the wing (C-S2) and (C) the folded zone of the costa (C-S3) of *C. japonicus* (10 ×), obtained using an inverted fluorescence microscope. The vein cavity is irregular.

Figure 3A–C demonstrates the cross sections of the wing base, the posterior part of the wing, and the folded zone in C, respectively, of the hind wings of *C. japonicus*, with cutting positons as shown in Figure 1D*.* The exocuticle, endocuticle, and epidermis layers appear brown and light pink (the thin basement membrane), respectively, in a manner similar to that found in [20]. It was shown that vein cavities are irregularly shaped, and their cross-section cavities get smaller and smaller. Figure 3A shows that the C+ScA cavity is thicker towards the dorsal than at the ventral part of the wing [20]. The area of the vein cavity in the posterior part of the wing is the smallest (Figure 3B), shown as a narrow strip. The end with wire-like objects is the wing membrane. The left cavity in Figure 3C is the C+ScA cavity, the middle is the R cavity and folded zone, and the right cavity is MP vein cavity. Due to the wing membrane being thin and having a certain toughness, slicing the veins will produce a certain extension, resulting in the observed length of the vein section being longer than the actual one. The cross section of the folded zone between R and MP only shows a thin wing membrane connection, which is related to the folding pattern and cutting position.

**Figure 4:**
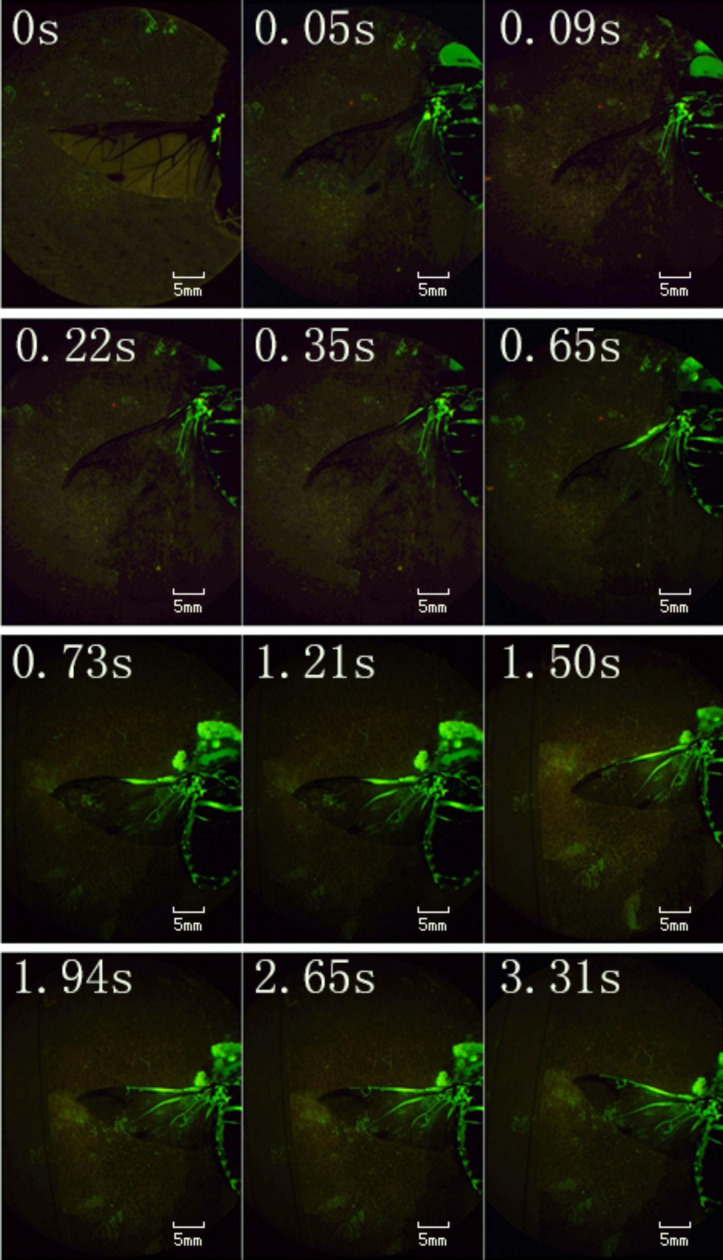
Fluorescence flow sequence in an unfolding hind wing of *C. japonicus*, captured using a retinal camera.

A retinal camera was used to confirm which veins were involved in the hydraulic mechanism of the unfolding of the hind wings. A fluorescent agent was injected into the abdomen of a live beetle. At 0.05 s, fluorescence was found in CuA, and at 0.09 s it nearly flowed through AAP; 1.21 s later, C, CuA, AAP, and AP_4_ were observed to be nearly full of fluorescence (Figure 4). After that, there was only a slow flow in C, and after 3.31 s, the fluorescence flowed through RA and stopped over 2 mm. This is different from what we observed in *Dorcus titanus platymelus*, in which the flow of body fluids is limited to the movement within the main veins – the costa and media posterior – and stops at the folding region [20]. This phenomenon is related to different profiles of C+ScA in the region of folding. Bending of the vein in *C. japonicus* is smooth in the folded wing, whereas in *Dorcus titanus platymelus* the turn is drastic. There, it is a sort of a hinge with a determinate axis of rotation, evident structure, and heterogenous structure: the anterior face of the bend is corrugated (a short bellows joint of 2–4 rings), the posterior face is a thin membrane, and the top and bottom faces are sclerotized plates. Pass [26] put forth the hypothesis that the hemolymph in some Coleoptera wings’ veins circulates in relation to periodic heartbeat reversal and intermittent pulse activity of the wing-hearts. Wasserthal [27] showed that injection of dye into the wings of *Pieris rapae* demonstrated that the movement of hemolymph into the wings along the veins was a unidirectional flow. In adult Lepidoptera, Coleoptera and Diptera, and perhaps in some other insects, the blood is shunted backwards and forwards between the thorax and abdomen, rather than circulated [1].

**Figure 5:**
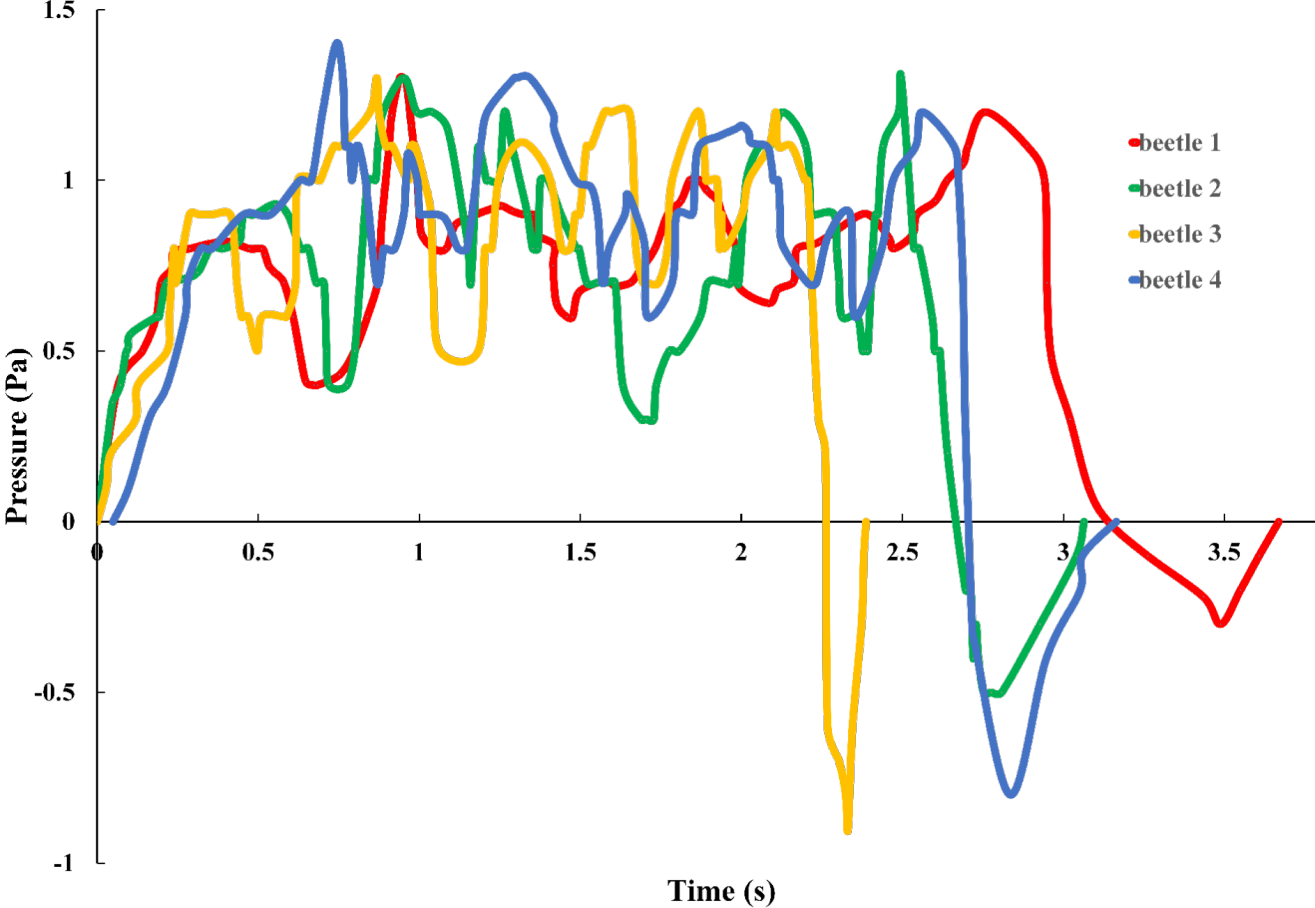
The change in blood pressure in the veins of the hind wings as a function of time.

**Figure 6:**
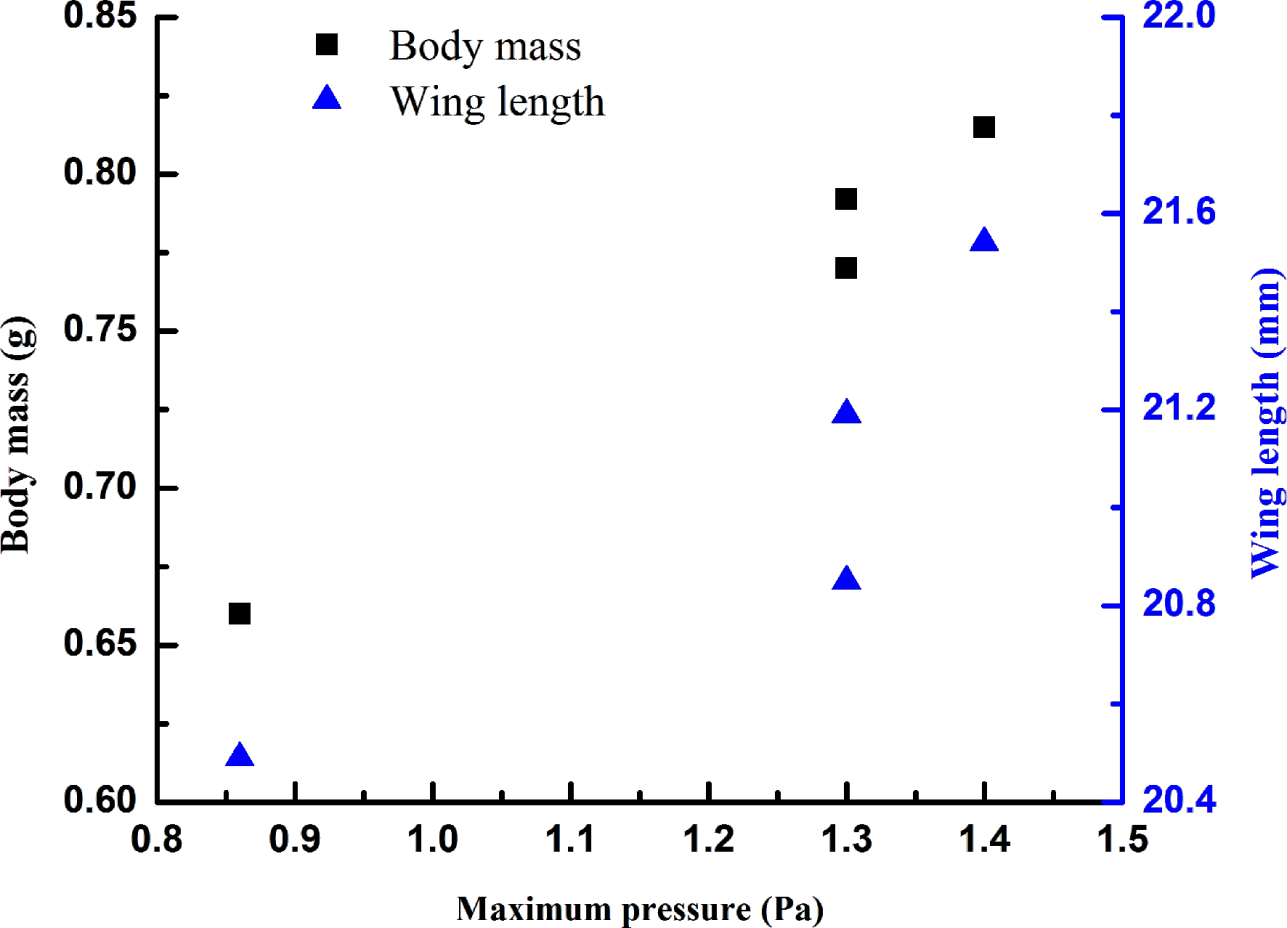
The blood pressure is proportional to the length of the wings and the body mass.

During the deployment of the hind wings, the blood pressure in the veins of the wings changes continuously (Figure 5): the pressure starts from zero, gradually and quickly increases to the peak, fluctuates over some time before decreasing to a minimum, and finally returning to zero when the actions of the beetle stop. This is different from the results described in [20]. The first peak pressure and time for the four beetles were (0.238 s, 0.8 Pa), (0.539 s, 0.9 Pa), (0.287 s, 0.9 Pa) and (0.322 s, 0.8 Pa). For beetle 4, at 0.742 s, there was a maximum peak pressure of 1.4 Pa, which made the hind wing fully expand after some time of fluctuation (alternating high and low pressures, 1.4 Pa, −1.08 Pa, −1.3 Pa, −0.96 Pa, −1.16 Pa, −0.9 Pa, −1.2 Pa), at 2.562 s, the pressure reached 1.2 Pa. Then, the pressure began to gradually decrease until, at 3.164 s, the pressure was 0 Pa with the hind wing completely extended. After this, when the hind wings were flapping, the pressure values indicated that there was a certain degree of flexibility due to the hydraulic energy in the presence of the hind wing, which helped stabilize the flight motion. Small pressure peaks of the blood and the hind wing being open appeared to overcome the remaining consistent folding. The expansion required a total time of 3.012 s to 3.670 s. This is due to the size of the pressure and the length and weight of the hind wings being proportional, as shown in Figure 6. It is possible that the wing was locked in the open position mechanically [9]. When the wing extensor or flexor muscles relax or contract, levers simultaneously open or close the wing, and surely the muscles and the blood pressure must operate synergistically. The negative pressures in Figure 5 could be the result of the abrupt muscular unfolding of the wing tip applying “suction” to the system. Another possible reason for the negative pressures could be that, before opening of the elytra, the prothorax is depressed relative to the pterothorax, thus unlocking the elytra. With the abdomen depressed synchronously with the opening of the elytra and wing flapping, the beetle elevated [23]. This elevation of the abdomen increased internal pressure in the body without influencing the unfolding.

This expansion time is a little quicker than that measured in Figure 4 (3.31 s). It is supposed that in that test (Figure 4), the beetle was fully expanded and fixed, while in this test (Figure 5), the beetle was able to move freely, which accelerated blood flow in a manner similar to that described in [20]. Moreover, this expansion time is slower than that measured in Figure 2 (approx. 0.741 s). One possible explanation is that the hind wing in that test is a record of a natural state and without any restraint; by contrast, the beetle in the pressure measurement (Figure 5) was connected to the sensor and had missing elytra.

### Simulation

The hexahedral mesh is orthogonal and gridded [28]. Thus, it will speed up convergence and improve the calculation accuracy. In Figure 1E, the total grid number shown in the graph is 135,000. At the same time, for the realization of the hind wing exercise, torsion was created in the folded position so that the fixed part and moving part of the costa veins were not in a plane (RA vein). Figure 1F and Figure 1G show the vein movement diagram. By using a high-speed camera to observe the folding/unfolding process of the beetle hind wings, it can be seen that the beetle costa vein root to the fold points is fixed (S1), and from the fold point to the end of the vein, where around their folds, inward folding occurred (S2 + S3). Accordingly, the costa was fixed to the first half; the rear section of the rotation movement around the respective rotation point, and specifically defined costa and RA vein of the center of rotation are the fold points. Unstructured meshes were used in the simulation; the computational domain was the internal domain. Inlet pressure was located in the root of the veins and exported to the wall for the single-phase flow model of computational physics. The open interface FLUENT allows users to customize the boundary conditions and other function variables. Thus, the pressure of the inlet was applied by the pressure boundary in this simulation, and a user-defined function (UDF) was used to apply the changing pressure implemented by the user program.

The blood circulation of insects is pumped by the heart in the abdomen, with arteries flowing to the head [1]. Because the front of the blood pressure is higher, it forces blood flow to the back of the blood chamber so that blood circulates in the entire blood chamber. The back side of the blood pressure in the accessory palsatory organ occurs through blood transmission to the outer epidermis of the vein because the liquid can transfer pressure in any direction, resulting in an axis of rotation of the torque and veins caused by rotation.

**Figure 7:**
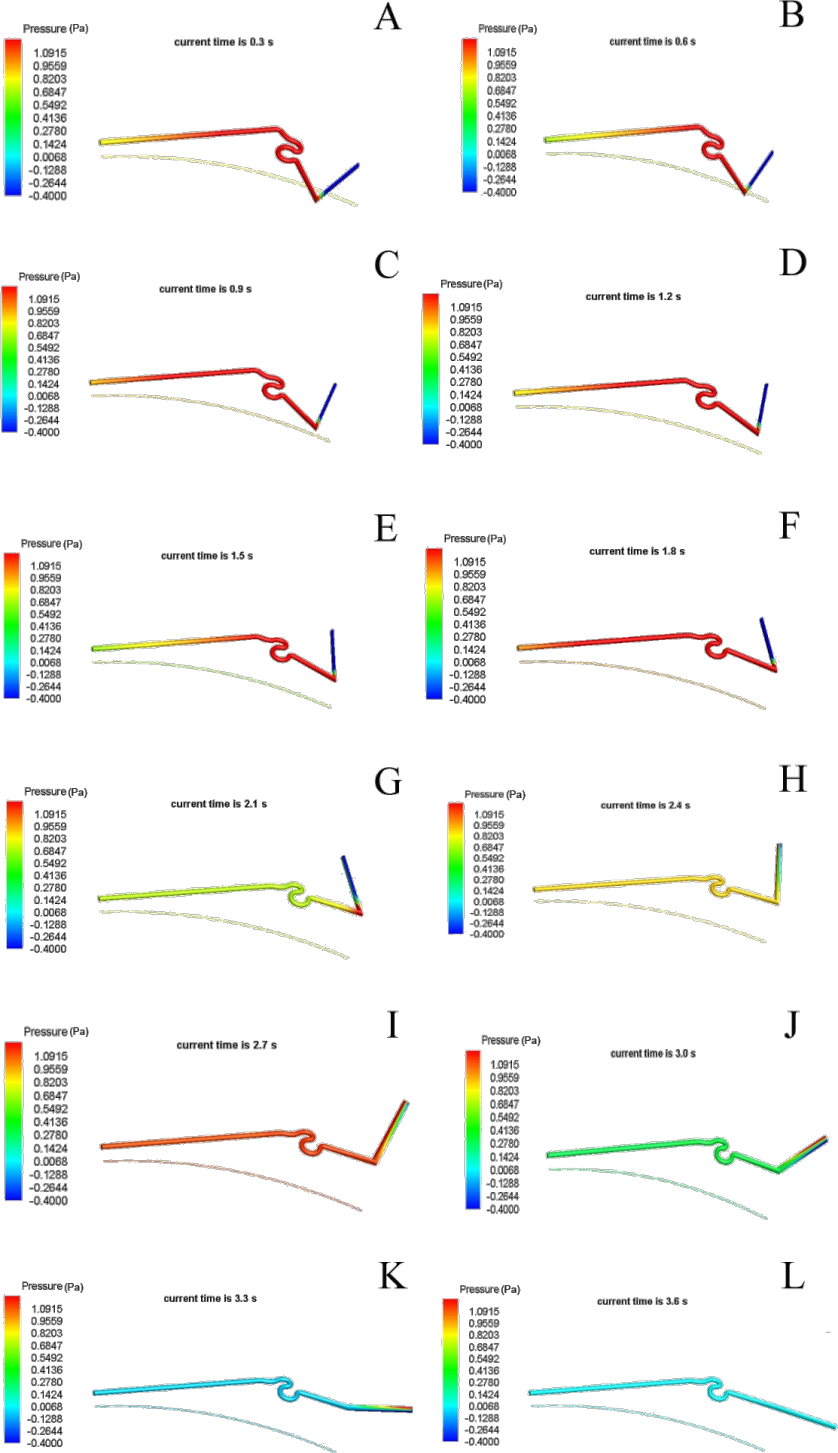
The simulation results of static pressure in a vein of a hind wing.

There are two stages: in the first stage, S1 is fixed and S2 rotates around the point q1; in the second stage, S1 and S2 are fixed, and S3 rotates around the point q2. As shown in Figure 7A–F, due to the relatively large geometric angle of the turning point, the blood will have a direct impact on the vein wall. Thus, in the formation of high pressure, especially in the first stage of the movement, the pressure is very obvious. In the first stage, at 0.9 s, the pressure in the blood vessel is more than 1 Pa because of the difference in pressure between the inside and outside of the walls of blood vessels, thus forming a rotating point and rotating torque. After 2.1 s, the first stage stops, and the middle part of the vein does not move. The pressure difference concentrates on the rotating point of the posterior segment, and there is the second moment of rotation. When S2 is completely extended around q1, the pressure in S1 is reduced, and high pressure concentrates in the rotated point q2 (2.1 s, Figure 7G). By this pressure, S3 is rotated by a little angle (2.4 s, Figure 7H). Then, the pressure continues to increase (2.7 s, Figure 7I), and at 3.0 s, the pressure in the veins undergoes a sharp decline until the end of the movement (Figure 7J–L). The pressure is greater than the end of the blood vessel vascular segment, which is mainly due to export to the wall; the flow will be stagnant here, and thus a high pressure zone will form. The high pressure area produces a torque around the rotation point. In the second stage, due to S1 and S2 being kept fixed, the differential pressure between the folding point and the entrance is obviously reduced. At the same time, to rotate S3, the differential pressure between the rotating point and export is larger. Between 3.3 and 3.6 s, the pressure differential of the venation front and end gradually decrease due to the venation almost having motion toward the limit position and the pressure transmission occurring more smoothly, thus producing reduced torque and slower speed.

**Figure 8:**
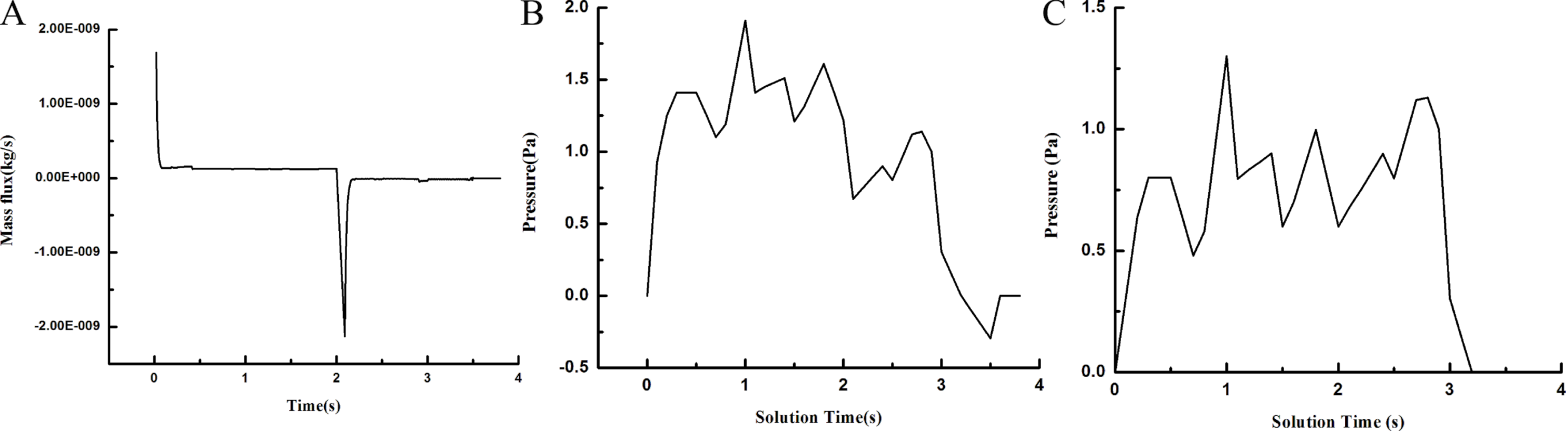
Blood flow changes in the venation of a hind wing of *C. japonicus* at the entrance (A); pressure change as a function of the time at rotation points q1 (B) and q2 (C).

The flow changes in the vasculature were also simulated (Figure 8). Figure 8A shows the change of flow as a function of the time (minus denotes outflow) in the wing base of the C vein (entrance point). Because the density is constant, the flow change directly responds to the variation in the flow velocity. At the beginning (0.1 s), because the entrance pressure is high, the hind wing venation just begins to unfold, with a great deal of blood flow from the wing base to the veins. With the movement gradually accelerated and the extending angle gradually increased, the veins produce a negative effect on the blood in the extending process of the wing, leading to outflow from the entrance. There is a slight fluctuation until the veins are fully expanded, and the entrance flow tends to 0; Figure 8B and Figure 8C show the pressure change with time at q1 and q2, similar to Figure 5 (the actual test curve).

Via FLUENT post-processing, we set up monitoring points at q1 and q2. In Table 1, before 2 s, the pressure loss of q1 is larger, which is due to the larger relative movement that exists in the left side and right side of q1 in stage 1. Thus, the relative flow and pressure loss are higher. After 2 s, the pressure loss of q1 is rapidly reduced to 0, which is due to q1 being in a static state in the second movement stage. Thus, there is no pressure loss. Similarly, for q2, the pressure loss always exists because it keeps moving.

**Table 1:** Pressure loss of flow during unfolding of the hind wing.

time (s)	pressure loss in q1 (Pa)	pressure loss in q2 (Pa)

0.6	0.608	0.610
1.2	0.606	0.607
1.8	0.904	0.905
2.4	0.001	0.028
3.0	0.0	0.005
3.6	0	0

## Conclusion

The unfolding of the hind wings of *C. japonicus* and its hydraulic mechanism were investigated. To confirm which veins were involved in the hydraulic mechanism of the unfolding of the hind wings, the cross sections of the veins were captured using an inverted fluorescence microscope and flow of blood was examined by means of a retinal camera. After 3.31 s, C, CuA, AAP, and AP_4_ were observed to be nearly full of fluorescence, and the fluorescence stopped at over RA 2 mm. To determine the unfolding action of the hind wings and corresponding time required for each action during the unfolding process, high speed camera sequences were used (400 frames/s). It was found that the beetle can start to flap its hind wings while they are still folded (0.651 s), and until 0.741 s, the hind wings were completely extended. Then, the pressure of the vein of the hind wings of four beetles during the unfolding process was tested. The results showed that with the unfolding action of the hind wings, the pressure kept changing. The wing veins unfolded within the flow field successive dynamic simulation deployment process of the hind wing during the entire time of travel in FLUENT. This is mainly from the two aspects of pressure and the velocity vector of the corresponding numerical study on the movement characteristics, which provide a bionic foundation to design and develop flying micro-robots.
